# Clinical Presentation, Risk Factors and Outcome of Non-Tuberculous Mycobacteria Infection in Hematopoietic Stem-Cell Transplantation: A Multinational Case-Control Study

**DOI:** 10.1093/ofid/ofag082

**Published:** 2026-02-19

**Authors:** Mario Fernández-Ruiz, Jose Tiago Silva, Peggy L Carver, Sasinuch Rutjanawech, Luis F Aranha-Camargo, Ruan Fernandes, Sara Belga, Amenah Alghamdi, Nicolas J Mueller, Sara Burkhard, Nicole M Theodoropoulos, Douwe F Postma, Pleun J van Duijn, Francisco Arnaiz de las Revillas, Concepción Pérez del Molino-Bernal, Jonathan Hand, Adam Lowe, Marta Bodro, Elisa Vanino, Ana Fernández-Cruz, Antonio Ramos-Martínez, Mateja Jankovic Makek, Ribal Bou Mjahed, Oriol Manuel, Antonia Calvo-Cano, Laura Rueda-Carrasco, Ana Álvarez-Uría, Regino Rodríguez-Álvarez, Alessandra Mularoni, Elisa Vidal, Teresa del Rosal, Yasmina Mozo, Annika Y Classen, Carlos Mejía-Chew, Francisco López-Medrano

**Affiliations:** Unit of Infectious Diseases, Hospital Universitario “12 de Octubre”, Instituto de Investigación Sanitaria Hospital “12 de Octubre” (imas12), School of Medicine, Universidad Complutense, Madrid, Spain; Centro de Investigación Biomédica en Red de Enfermedades Infecciosas (CIBERINFEC), Instituto de Salud Carlos III (ISCIII), Madrid, Spain; Unit of Infectious Diseases, Hospital Universitario “12 de Octubre”, Instituto de Investigación Sanitaria Hospital “12 de Octubre” (imas12), School of Medicine, Universidad Complutense, Madrid, Spain; Centro de Investigación Biomédica en Red de Enfermedades Infecciosas (CIBERINFEC), Instituto de Salud Carlos III (ISCIII), Madrid, Spain; Department of Pharmacy, University of Michigan College of Pharmacy, Ann Arbor, Michigan, USA; Department of Infectious Diseases, Washington University School of Medicine, St.Louis, Missouri, USA; Department of Infectious Diseases, Hospital Israelita Albert Einstein, São Paulo, Brazil; Department of Infectious Diseases, Hospital Israelita Albert Einstein, São Paulo, Brazil; Division of Infectious Diseases, Department of Medicine, University of British Columbia Faculty of Medicine, Vancouver, BC, Canada; Division of Infectious Diseases, Department of Medicine, King Abdulaziz University, Jeddah, Saudi Arabia; Department of Infectious Diseases, University Hospital Zurich, Zurich, Switzerland; Department of Infectious Diseases, University Hospital Zurich, Zurich, Switzerland; Department of Infectious Diseases, University of Massachusetts Chan Medical School, Worcester, Massachusetts, USA; Department of Internal Medicine and Infectious Diseases, University Medical Center Groningen, University of Groningen, Groningen, The Netherlands; Department of Clinical Microbiology, Certe Laboratory for Infectious Diseases,Groningen, The Netherlands; Centro de Investigación Biomédica en Red de Enfermedades Infecciosas (CIBERINFEC), Instituto de Salud Carlos III (ISCIII), Madrid, Spain; Department of Infectious Diseases, Hospital Universitario Marqués de Valdecilla, IDIVAL, Santander, Spain; Centro de Investigación Biomédica en Red de Enfermedades Infecciosas (CIBERINFEC), Instituto de Salud Carlos III (ISCIII), Madrid, Spain; Department of Infectious Diseases, Hospital Universitario Marqués de Valdecilla, IDIVAL, Santander, Spain; Department of Infectious Diseases, Ochsner Medical Center, New Orleans, Louisiana, USA; Department of Infectious Diseases, Ochsner Medical Center, New Orleans, Louisiana, USA; Centro de Investigación Biomédica en Red de Enfermedades Infecciosas (CIBERINFEC), Instituto de Salud Carlos III (ISCIII), Madrid, Spain; Department of Infectious Diseases, Hospital Clinic, IDIBAPS, Barcelona, Spain; Unit of Infectious Diseases, IRCCS Policlinico Sant’Orsola, University of Bologna, Bologna, Italy; Department of Internal Medicine, Unit of Infectious Diseases, Hospital Universitario Puerta de Hierro-Majadahonda, Instituto de Investigación Sanitaria Puerta de Hierro-Segovia de Arana (IDIPHISA), Majadahonda, Spain; Department of Internal Medicine, Unit of Infectious Diseases, Hospital Universitario Puerta de Hierro-Majadahonda, Instituto de Investigación Sanitaria Puerta de Hierro-Segovia de Arana (IDIPHISA), Majadahonda, Spain; Department for Pulmonary Diseases, School of Medicine, University Hospital Center Zagreb, University of Zagreb, Zagreb, Croatia; Department of Infectious Diseases, Lausanne University Hospital (CHUV) and University of Lausanne, Lausanne, Switzerland; Department of Infectious Diseases, Lausanne University Hospital (CHUV) and University of Lausanne, Lausanne, Switzerland; Department of Biomedical Sciences, Faculty of Medicine and Health Sciences, University Institute of Biosanitary Research of Extremadura (INUBE), Area of Medicine, University of Extremadura, Badajoz, Spain; Department of Biomedical Sciences, Faculty of Medicine and Health Sciences, University Institute of Biosanitary Research of Extremadura (INUBE), Area of Medicine, University of Extremadura, Badajoz, Spain; Department of Clinical Microbiology and Infectious Diseases, Hospital General Universitario Gregorio Marañón, Biomedical Research Institute Gregorio Marañon (IiSGM), Centro de Investigación Biomédica en Red de Enfermedades Respiratorias (CIBERES), Universidad Complutense, Madrid, Spain; Department of Infectious Diseases, Hospital Universitari de Cruces, Barakaldo, Spain; Department of Infectious Diseases, Istituto Mediterraneo per i Trapianti e Terapie ad Alta Specializzazione (IRCC-ISMETT), Palermo, Italy; Centro de Investigación Biomédica en Red de Enfermedades Infecciosas (CIBERINFEC), Instituto de Salud Carlos III (ISCIII), Madrid, Spain; Department of Infectious Diseases, Hospital Universitario Reina Sofía, Universidad de Córdoba, Córdoba, Spain; Department of General Pediatrics and Infectious Diseases, Hospital Universitario La Paz, Hospital La Paz Institute for Health Research (IdiPAZ), Universidad Autónoma de Madrid, Center for Biomedical Network Research on Rare Diseases (CIBERER), Madrid, Spain; Department of General Pediatrics and Infectious Diseases, Hospital Universitario La Paz, Hospital La Paz Institute for Health Research (IdiPAZ), Universidad Autónoma de Madrid, Center for Biomedical Network Research on Rare Diseases (CIBERER), Madrid, Spain; Department I for Internal Medicine, Faculty of Medicine and University Hospital Cologne, University of Cologne, Cologne, Germany; German Centre for Infection Research (DZIF), Partner Site Bonn-Cologne, Cologne, Germany; Department of Infectious Diseases, Washington University School of Medicine, St.Louis, Missouri, USA; Unit of Infectious Diseases, Hospital Universitario “12 de Octubre”, Instituto de Investigación Sanitaria Hospital “12 de Octubre” (imas12), School of Medicine, Universidad Complutense, Madrid, Spain; Centro de Investigación Biomédica en Red de Enfermedades Infecciosas (CIBERINFEC), Instituto de Salud Carlos III (ISCIII), Madrid, Spain

**Keywords:** case-control study, hematopoietic stem-cell transplantation, non-tuberculous mycobacteria, risk factors, treatment

## Abstract

**Background:**

The clinical and microbiological features of infection due to non-tuberculous mycobacteria (NTM) after hematopoietic stem-cell transplantation (HSCT) remain poorly understood.

**Methods:**

We performed a retrospective, multinational case-control study that included HSCT recipients (≥12 years) diagnosed with NTM disease between January 2008 and December 2018. Controls were HSCT recipients with no evidence of NTM disease, matched (1:2 ratio) by participating center and post-transplant survival. Logistic regression on matched pairs was used to investigate risk factors for NTM disease.

**Results:**

We included 25 cases of NTM disease. The most common HSCT type was allogeneic from unrelated donor (72.0%) after myeloablative conditioning (76.0%). Predominant hematological conditions were acute myelogenous leukemia (28.0%) and myelodysplastic syndrome (24.0%). Most patients (88.0%) had previously received immunosuppressive therapy. The most common species identified were *Mycobacterium avium* complex (64.0%) and rapidly growing mycobacteria (20.0%). Most patients (68.0%) had pulmonary disease. All but one received antimycobacterial therapy for a median of 267.5 days. Macrolides (83.3%), rifamycins (58.3%) and ethambutol (62.5%) were the most commonly used drugs. Four patients (16.7%) developed adverse events requiring therapy discontinuation. All-cause and attributable mortality rates were 28.0% and 4.0%, respectively. One patient experienced relapse after 464 days. Diagnosis of a non-NTM infection (adjusted odds ratio [aOR]: 3.11; 95% confidence interval [95% CI]: 1.25–7.78) and corticosteroid therapy (aOR: 2.88; 95% CI: 1.16–7.17), both within the previous 90 days, were associated with NTM disease.

**Conclusions:**

NTM disease is a serious complication among heavily immunocompromised HSCT recipients associated with prior non-NTM infection and corticosteroid therapy.

## Teaser

In this multicenter case–control study, we investigated the clinical, radiological, and microbiological characteristics of non-tuberculous mycobacterial (NTM) infection in hematopoietic stem-cell transplant recipients, as well as associated risk factors and clinical outcomes. A prior diagnosis of non-NTM infection and the use of corticosteroid therapy within the preceding 90 days were identified as factors associated with the development of this rare but potentially severe complication.

## Graphical Abstract

**Graphical Abstract:**
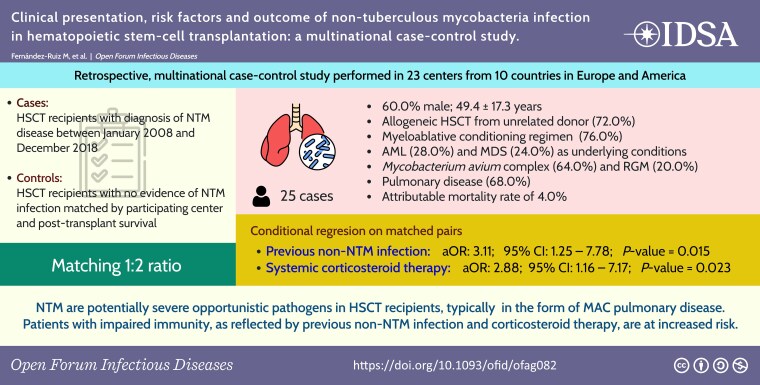
This graphical abstract is also available at Tidbit: https://tidbitapp.io/tidbits/clinical-presentation-risk-factors-and-outcome-of-non-tuberculous-mycobacteria-infection-in-hematopoietic-stem-cell-transplantation-a-multinational-case-control-study?utm_campaign=tidbitlinkshare&utm_source=IO

Non-tuberculous mycobacteria (NTM) are increasingly recognized as relevant pathogens in older individuals with chronic structural lung conditions and in immunocompromised patients. This trend may be in part explained by environmental factors, improvements in diagnostic methods and higher clinical awareness [1]. The publication of clinical guidelines and consensus recommendations—including the proposal of diagnostic criteria for pulmonary disease—has constituted a major contribution to the management of NTM infection [2–5]. Nevertheless, this entity continues to be challenging for the physicians due to its low incidence, broad clinical spectrum, heterogeneous virulence across NTM species, difficulties in distinguishing between true disease and colonization, common resistance to many of the antimycobacterial drugs, and frequency of adverse events (AEs) during treatment [6].

Since protective immunity against mycobacteria primarily relies on interferon-γ-producing T-cell-mediated Th1 responses [7], it is not surprising those hematopoietic stem-cell transplantation (HSCT) acts as a predisposing factor for the development of NTM disease [8]. Allogeneic HSCT (allo-HSCT) recipients usually show impaired T-cell-mediated immunity due to the cumulative effect of the underlying hematological disease, pre-transplant conditioning regimen, *ex vivo* T-cell depletion protocols, and graft-versus-host disease (GVHD) prophylaxis and treatment [9]. On the other hand, patients with lymphoma or multiple myeloma undergoing high-dose chemotherapy followed by autologous HSCT (auto-HSCT) often experience delayed T-cell reconstitution that may persist for at least 1 year [10, 11]. Once considered an exceedingly uncommon event (with incidence rates below 1% in early series [12, 13]), NTM disease has been found to occur in 1% to 5% of allo-HSCT recipients in more recent studies, depending on the geographical region [14–16]. This incidence is 50-to-fold higher than in the general population [17]. On the other hand, the prognosis is often dismal, with reported attributable mortality rates exceeding 30% [18].

To identify the patients at increased risk of NTM disease, a better understanding of the clinical presentation and predisposing factors is needed. The available literature, however, is limited, with most studies based on single-center experiences collected over long periods of time [16, 19–23] or restricted to pediatric patients [9] or specific sites of infection [15]. Few studies have investigated specific risk factors in this population [16, 21, 23], and most of them were performed in high-endemic areas [16, 23], which limited the generalizability of the results.

In this multinational case-control study, we assessed the clinical and microbiological features and outcomes of NTM disease in a representative sample of HSCT recipients from a large number of centers worldwide, as well as the risk factors for developing this complication.

## PATIENTS AND METHODS

### Study Design and Setting

We have performed an analysis of HSCT recipients diagnosed with NTM disease between January 2008 and December 2018 in the EMOTE Collaboration, a multinational retrospective case-control study that also included solid organ transplant (SOT) recipients [24, 25]. Both autologous and allogeneic procedures were included. Overall, 23 centers from 10 countries in Europe and America contributed cases and controls. All sites followed national research regulations and obtained approval from their local review boards, as required. The requirement for informed consent was waived due to the retrospective design and information was collected in compliance with the European Union General Data Protection Regulation and the 1996 US Health Insurance Portability and Accountability Act.

We included allo- and auto-HSCT recipients aged 12 years or older that met the case definition for NTM disease during the post-transplant follow-up period (cases). For each case included two HSCT recipients with no evidence of NTM infection and matched by participating center and post-transplant survival (to equal the time elapsed from transplant to diagnosis of NTM disease of the corresponding case) were recruited (controls).

NTM species identification could be based on different methods according to local practices in each center: biochemical tests (typically for cases diagnosed in the first years of the study period), nucleic acid probes, PCR-restriction enzyme pattern analysis or matrix-assisted laser desorption ionization-time of flight (MALDI-TOF) mass spectrometry (for the most recent cases). Antimycobacterial susceptibility testing was performed according to the applicable edition of the Clinical and Laboratory Standards Institute (CLSI) guidelines. Episodes of infection due to pathogens other than NTM (non-NTM infection) were diagnosed on the basis of clinical and routine microbiological data.

### Study Definitions and Data Collection

The definition of NTM disease (pulmonary or extrapulmonary) was established according to the clinical, imaging and microbiological diagnostic criteria recommended by the guidelines in place during the study period [2, 26]. The diagnosis was also assumed if the hematologist or infectious diseases physician caring for the patient prescribed antimycobacterial treatment for at least 2 weeks upon a positive culture for NTM that was consistently deemed clinically relevant. Disseminated NTM disease required the involvement of two or more noncontiguous organs. The date of diagnosis was based on the calendar date on which the first positive sample(s) meeting case definition were collected. Controls were assigned a “pseudodate” of diagnosis to match the post-transplant follow-up of their corresponding cases. Neutropenia and lymphopenia were defined by absolute neutrophil and lymphocyte counts <0.5 × 10^9^ cells/L. Hypogammaglobulinemia was defined as a serum IgG level <700 mg/dL.

We used a standardized case report form to collect the following data from electronic or paper-based health records by local researchers at participating centers: demographics; comorbidities (within the year prior to diagnosis); hematological disease; type of HSCT; conditioning regimen; complications following HSCT (requirement for intensive care unit [ICU] admission, mechanical ventilation or renal replacement therapy, cytomegalovirus [CMV] disease); diagnosis of GVHD; diagnosis of other non-NTM infection requiring hospital admission (within the 90 days prior to diagnosis); receipt of immunosuppressive drugs, chemotherapy or radiotherapy in the previous 90 days; and laboratory data closest to the index date. All the patients were followed-up for at least 2 years from the date of diagnosis (or the “pseudodate” for controls), unless death occurred earlier.

For NTM disease cases, we also collected information on clinical symptoms and radiological features at presentation, microbiological variables (NTM species, smear positivity, pathological findings, time to culture positivity, number of positive cultures, type of specimen and *in vitro* susceptibility), antimycobacterial therapy (agents, duration, treatment-related AEs requiring discontinuation of therapy), surgical resection, and patients' outcomes (mortality and cause of death).

Attributable mortality was defined as death occurring with persistent signs and symptoms of NTM disease, positive cultures and/or while on antimycobacterial therapy with no alternative cause identified, as established by the treating physician. All-cause mortality was defined as death from any cause occurring later than the date of diagnosis of NTM disease (or the “pseudodate” for controls).

Data were collected and managed using the secure web-based software platform REDCap (Research Electronic Data Capture, Vanderbilt University) hosted at the Washington University in St. Louis, Missouri, USA.

### Statistical Analysis

Categorical variables were compared using the χ^2^ test or Fisher exact test, whereas Student's t-test or Mann-Whitney U test were applied for continuous variables. Conditional logistic regression on matched pairs was used to identify risk factors for the development of NTM disease. Those variables found to be significant (*P*-value ≤.05) at the univariable analysis were included into the multivariable model. Collinearity among variables was assessed by variance inflation factors (VIFs). Statistical analysis was performed using SPSS version 20.0 (IBM Corp., Armonk, NY).

## RESULTS

### Clinical Presentation and Microbiological Features of NTM Disease after HSCT

We included 25 HSCT recipients diagnosed with NTM disease during the study period. Individual patient-level data on clinical presentation, radiological features and outcomes are detailed in Supplementary Table S1. The median age at diagnosis was 56.5 years (IQR: 30.8–61.9), with male predominance (15 [60.0%]). The most common type of HSCT was allogeneic from an unrelated donor (18 [72.0%]) after myeloablative conditioning (19 [76.0%]). Predominant underlying hematological conditions were acute myelogenous leukemia (AML) (7 patients [28.0%]) and myelodysplastic syndrome (MDS) (6 [24.0%]). Most patients had GVHD (21 [84.0]) and had received systemic immunosuppression in the previous 90 days (22 [88.0%]) (Table 1).

**Table 1.** Demographics, Comorbidities, Predisposing Factors and Underlying Conditions of HSCT Recipients with NTM disease (*n* = 25)

Variable	…
Age at diagnosis, years [median (IQR)]	56.5 (30.8–61.9)
Male gender [*n* (%)]	15 (60.0)
Comorbidities [*n* (%)]^a^	…
Chronic lung condition	7 (28.0)
COPD	4 (16.0)
Other^b^	3 (12.0)
Hypertension	8 (32.0)
Diabetes mellitus	5 (20.0)
Gastroesophageal disease	5 (20.0)
Obesity (BMI ≥30 Kg/m^2^)	3 (12.0)
Autoimmune disease	3 (12.0)
Chronic renal disease	2 (8.0)
End-stage liver disease	1 (4.0)
Previous NTM colonization [*n* (%)]	3 (12.0)
Region of origin [*n* (%)]	…
North America	16 (64.0)
Western Europe	6 (24.0)
South America	1 (4.0)
North Africa	1 (4.0)
Eastern Asia	1 (4.0)
Infections other than NTM disease [*n* (%)]	17 (68.0)
Pneumonia (non-opportunistic pathogen)^c^	8 (32.0)
Sepsis (non-opportunistic pathogen)^c^	5 (20.0)
Non-CMV opportunist infection^c,d^	5 (20.0)
CMV disease^e^	3 (12.0)
Other non-opportunistic infection^c,f^	6 (24.0)
Active chemotherapy^c^	5 (20.0)
Parenteral nutrition^c^	3 (12.0)
ICU admission^c^	5 (20.0)
Type of HSCT [*n* (%)]	…
Related allogeneic	18 (72.0)
Unrelated allogeneic	5 (20.0)
Autologous	2 (8.0)
Conditioning regimen [*n* (%)]	…
Myeloablative	19 (76.0)
Non-myeloablative/reduced-intensity	6 (24.0)
Underlying hematological condition [*n* (%)]	…
AML	7 (28.0)
MDS	6 (24.0)
B-cell leukemia or lymphoma	5 (20.0)
MM	2 (8.0)
Other^g^	2 (8.0)
Non-malignant condition^h^	3 (12.0)
Relapsed disease after HSCT [*n* (%)]	3 (12.0)
GVHD [*n* (%)]	21 (84.0)
Immunosuppressive therapy [*n* (%)]^c^	22 (88.0)
Calcineurin inhibitor	14 (56.0)
Ruxolitinib	6 (24.0)
Mycophenolate mofetil	3 (12.0)
mTOR inhibitor	2 (8.0)
Etanercept	2 (8.0)
Systemic corticosteroids	17 (68.0)
Daily prednisone-equivalent dose, mg [median (IQR)]	12.5 (5–25)

AML, acute myelogenous leukemia; BMI, body mass index; CMV, cytomegalovirus; COPD, chronic obstructive pulmonary disease; GVHD, graft-versus-host disease; HSCT, hematopoietic stem-cell transplantation; ICU, intensive care unit; IQR, interquartile range; MDS, myelodysplastic syndrome; MM, multiple myeloma; NTM, non-tuberculous mycobacteria; SD, standard deviation.

^a^Within the year prior to diagnosis.

^b^Includes bronchiectasis, bronchiolitis obliterans and lymphocytic interstitial pneumonia (one case each).

^c^Within 90 days prior to diagnosis.

^d^Includes invasive pulmonary aspergillosis, *Candida* esophagitis, nocardiosis, BK virus-associated hemorrhagic cystitis and disseminated adenovirus infection (one case each).

^e^Within 180 days prior to diagnosis.

^f^Includes catheter-associated BSI due to coagulase-negative staphylococci (two cases), acute pyelonephritis, intraabdominal infection, soft and soft tissue infection and osteomyelitis (one case each).

^g^Includes chronic myelogenous leukemia and blastic plasmacytoid dendritic cell neoplasm.

^h^Includes aplastic anemia, common variable immunodeficiency and CTLA-4 deficiency (one case each).

The median interval from transplantation to diagnosis was 506.5 days (interquartile range [IQR]: 152–1884.5). The most common sites of NTM disease were pulmonary (17 [68.0%]) and catheter-related bloodstream infection (BSI) (3 [12.0%]). There were two cases (8.0%) of disseminated disease. Cough (11 [44.0%]), dyspnea (10 [40.0%]) and fever (9 [36.0%]) were the predominantly reported symptoms at presentation. Although neutropenia was uncommon (2 [9.1%]), lymphopenia was present in more than one-third of patients with available data (8 [36.4%]). In addition, almost half of evaluable patients had IgG hypogammaglobulinemia (8/17 [47.1%]) (Table 2).

**Table 2.** Clinical Presentation and Laboratory Values of Cases of NTM Disease (*n* = 25)

Variable	…
Time interval from HSCT to diagnosis, days [median (IQR)]	506.5 (152–1884.5)
Symptoms at diagnosis [*n* (%)]	…
Cough	11 (44.0)
Dyspnea	10 (40.0)
Fever	9 (36.0)
Fatigue	8 (32.0)
Weight loss	3 (12.0)
Lymphadenopathy	1 (4.0)
Massive hemoptysis	1 (4.0)
Time interval from symptoms onset to diagnosis, days [median (IQR)]	15 (7–60)
Site of NTM disease [*n* (%)]	…
Pulmonary only	17 (68.0)
Non-pulmonary	6 (24.0)
Catheter-related BSI	3 (12.0)
Cutaneous	2 (8.0)
Lymphadenitis	1 (4.0)
Disseminated disease	2 (8.0)
Laboratory values at diagnosis^a^	…
WBC count, ×10^9^ cells/L [median (IQR)]	5.3 (2.0–6.9)
ANC, ×10^9^ cells/L [median (IQR)]	3.2 (1.4–5.3)
Neutropenia [*n* (%)]	2 (9.1)
ALC, ×10^9^ cells/L [median (IQR)]	0.7 (0.4–1.0)
Lymphopenia [*n* (%)]	8 (36.4)
Hemoglobin, g/dL [mean ± SD]	10.4 ± 1.8
Platelet count, ×10^9^ cells/L [median (IQR)]	125 (44.3–278.3)
Serum creatinine, mg/dL [mean ± SD]	0.9 ± 0.3
Serum glucose, mg/dL [mean ± SD]	109.8 ± 33.9
LDH, IU/L [median (IQR)]	327 (181–460)
AST, IU/L [median (IQR)]	30.5 (25–45)
ALT, IU/L [median (IQR)]	26.5 (15.8–46.3)
Alkaline phosphatase, IU/L [median (IQR)]	111 (79–169)
Total bilirubin, mg/dL [median (IQR)]	0.5 (0.4–0.7)
Immunoglobulin G, mg/dL [median (IQR)]^b^	711 (561–847.5)
Hypogammaglobinemia [*n* (%)]	8/17 (47.1)

ALC, absolute lymphocyte count; ALT, alanine aminotransferase; ANC, absolute neutrophil count; AST, aspartate aminotransferase; BSI, bloodstream infection; HSCT, hematopoietic stem-cell transplantation; IQR, interquartile range; LDH, lactate dehydrogenase; NTM, non-tuberculous mycobacteria; SD, standard deviation; WBC, white blood cell.

^a^Laboratory data were not available for 3 patients.

^b^Data on serum immunoglobulin G levels were not available for 8 patients.

Most cases (17 [68.0%]) were diagnosed on the basis of specimens obtained from the respiratory tract (bronchoalveolar lavage [BAL], bronchial aspirate and/or transbronchial biopsy). Polymerase chain reaction for NTM was performed in only one case (BAL sample), with a negative result. The most common findings on thoracic computed tomography (CT) scan were nodules (9/19 [47.4%]) and interstitial infiltrates (8/19 [42.1%]), whereas the presence of bronchiectasis (3/19 [15.8%]) or cavitation (1/19 [5.3%]) was rare (Table 3).

**Table 3.** Microbiological and Radiological Features of Cases of NTM Disease (*n* = 25)

Variable	…
Number of positive cultures with isolation of the same NTM species [median (IQR)]	1 (1–2)
Smear positivity [*n* (%)]	9 (36.0)
Type of specimen [*n* (%)]^a^	…
BAL, bronchial aspirate and/or transbronchial biopsy	17 (68.0)
Sputum	4 (16.0)
Skin	3 (12.0)
Blood	3 (12.0)
Bone marrow	1 (4.0)
Lymph node	1 (4.0)
PCR for NTM performed [*n* (%)]	1 (4.0)
Pathological examination [*n* (%)]	12 (48.0)
Type of biopsy specimen	…
Transbronchial biopsy	7 (28.0)
Skin	2 (8.0)
Bone marrow	1 (4.0)
Lymph node	1 (4.0)
Soft tissue	1 (4.0)
Positive AFB smear^b^	4/12 (33.3)
Granulomatous inflammation^b^	4/12 (33.3)
Chest X ray performed at diagnosis [*n* (%)]	19 (76.0)
Radiological findings^b^	…
Interstitial infiltrates	6/19 (31.6)
Alveolar infiltrates	5/19 (26.3)
Nodules	2/19 (10.5)
Cavitation	1/19 (5.3)
Bronchiectasis	1/19 (5.3)
Thoracic CT scan performed at diagnosis [*n* (%)]	19 (76.0)
Radiological findings^b^	…
Nodules	9/19 (47.4)
Interstitial infiltrates	8/19 (42.1)
Alveolar infiltrates	5/19 (26.3)
Bronchiectasis	3/19 (15.8)
Pleural effusion	2/19 (10.5)
Cavitation	1/19 (5.3)

AFB, acid-fast bacilli BAL, bronchoalveolar lavage; CT, computed tomography; IQR, interquartile range; NTM, non-tuberculous mycobacteria; PCR, polymerase chain reaction.

^a^Relative frequencies sum more than 100.0% because some patients had more than one positive result.

^b^Percentage calculated on the number of patients in which the corresponding diagnostic method was performed.

**Figure 1.**
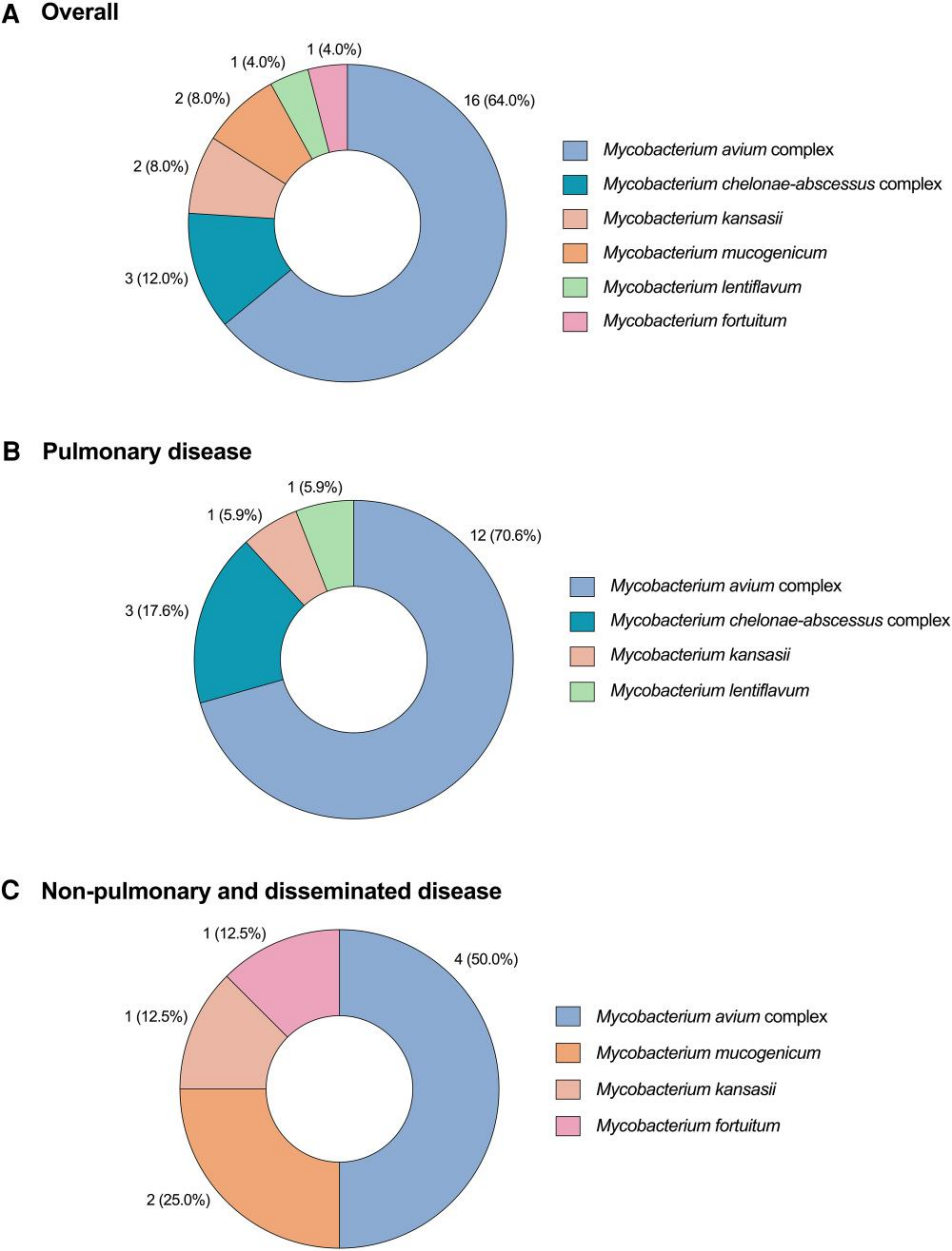
NTM species distribution: (A) overall cohort (*n* = 25); (B) cases with pulmonary disease only (*n* = 17); (C) non-pulmonary or disseminated disease (*n* = 8). Data are expressed as absolute numbers (percentages).

Regarding species distribution, most cases were caused by the *Mycobacterium avium* complex (MAC) (16 [64.0%]), followed by the *M. chelonae-abscessus* complex (3 [12.0%]). Three out of 16 cases (18.8%) of MAC infection were due to *M. chimaera*. Rapidly growing mycobacteria (RGM) were identified in one-fifth of the cases (5 [20.0%]). According to the site of disease, the predominance of MAC was more evident in cases with pulmonary involvement only, as compared to non-pulmonary or disseminated disease (12/17 [70.6%] versus 4/8 [50.0%], respectively) (Figure 1). *In vitro* antimycobacterial susceptibly was evaluated by the CLSI methodology in 20 cases (76.9%). As detailed in Supporting Material (Supplementary Table S2), all MAC isolates were susceptible to macrolides.

### Therapeutic Approaches

Most patients (24 [96.0%]) received antimycobacterial therapy upon diagnosis of NTM disease, for a median of 267.5 days (IQR: 29.8–427). The classes most commonly administered were macrolides (20/24 [83.3%]), rifamycins (14/24 [58.3%]), ethambutol (15/24 [62.5%]) and fluoroquinolones (8/24 [33.3%]). Patients were treated with a median of 3 (IQR: 3–4) different antimycobacterial drugs, and the most frequent combination was rifamycins, macrolides and ethambutol (11/24 [45.8%]). The length of therapy was shorter in cases of BSI as compared to the rest of the cohort (median of 29 versus 291 days, respectively; *P*-value = .052).

Four patients (16.7%) experienced at least one AE requiring therapy discontinuation: ototoxicity with amikacin (*n* = 2), gastrointestinal toxicity with ethambutol (*n* = 1), leukopenia with rifampicin (*n* = 1), leukopenia, thrombocytopenia and peripheral neuropathy with linezolid (*n* = 1), and tendinopathy with levofloxacin (*n* = 1) (Table 4).

**Table 4.** Therapeutic Approaches and Outcomes of Cases of NTM Disease (*n* = 25)

Variable	…
Receipt of antimycobacterial therapy [*n* (%)]	24 (96.0)
Duration of therapy, days [median (IQR)]	267.5 (29.8–427)
Time interval from diagnosis to initiation of therapy, days [median (IQR)]	22 (8–46)
Number of antimycobacterial drugs [median (IQR)]	3 (3–4)
Antimycobacterial drug [*n* (%)]	…
Macrolide	20/24 (83.3)
Ethambutol	15/24 (62.5)
Fluoroquinolone	8/24 (33.3)
Rifampicin	8/24 (33.3)
Rifabutin	6/24 (25.0)
Amikacin	4/24 (16.7)
Carbapenem	3/24 (12.5)
Tigecycline	2/24 (8.3)
Other	6/24 (25.0)
Treatment-related AE requiring discontinuation of the antimycobacterial drug [*n* (%)]	4/24 (16.7)
Time interval from initiation of drug to discontinuation, days [median (IQR)]	34 (16–93)
Reduction or withdrawal of immunosuppression [*n* (%)]	5/22 (22.7)
Surgical treatment [*n* (%)]	2 (8.0)
Evaluation by an ID physician [*n* (%)]	19 (76.0)
Evaluation by a pulmonologist [*n* (%)]	11 (44.0)
Outcomes	…
Time interval to the first respiratory sample with negative NTM culture, days [median (IQR)]^a^	30 (2–227)
New positive culture after previous culture negativization [*n* (%)]	3 (12.0)
Relapse of NTM disease [*n* (%)]	1 (4.0)
All-cause mortality [*n* (%)]	7 (28.0)
One-year mortality^b^	4/24 (16.7)
Two-year mortality^b^	7/24 (29.2)
Attributable mortality [*n* (%)]	1 (4.0)

AE, adverse event; ID, infectious diseases; IQR, interquartile range.

^a^Data on the time interval to the first negative NTM culture was not available for 2 patients.

^b^Mortality rates calculated on the basis of the number of patients with a minimum 1 and 2-year follow-up.

The median follow-up for cases of NTM disease was 8.1 years (IQR: 5.6–11.8). All-cause and attributable mortality rates were 28.0% (7/25) and 4.0% (1/25), respectively. In detail, one patient died in the setting of disseminated *M. kansasii* infection with pulmonary and cutaneous involvement 4 months after diagnosis. On the other hand, one patient (4.0%) experienced relapse of NTM disease due to MAC after 464 days from the discontinuation of therapy.

### Risk Factors for NTM Disease

A second appropriate control was not found for two of the cases. Therefore, the analysis of risk factors for the development of NTM disease was performed on the basis of 23 cases and 46 controls matched (1:2 ratio) by center and survival time after HSCT. There were no significant differences in all-cause mortality between cases and controls (30.4% [7/23] versus 30.4% [14/46], respectively; *P*-value = 1.000).

In the univariate analysis, the following factors were associated with the development of NTM disease: prior diagnosis of a chronic lung condition, infection other than NTM disease within the previous 90 days, requirement of ICU admission within the previous 90 days, unrelated allogeneic (versus related allogeneic or autologous) HSCT, and treatment with ruxolitinib and systemic corticosteroids within the previous 90 days. The variables of “previous ICU admission” and “ruxolitinib treatment” showed some evidence of collinearity (VIF value >1.1) and were excluded from the multivariable analysis to avoid model overinflation and overfitting.

Finally, the previous non-NTM infection (adjusted odds ratio [aOR]: 3.11; 95% confidence interval [CI]: 1.25–7.78; *P*-value = .015) and systemic corticosteroid therapy (aOR: 2.88; 95% CI: 1.16–7.17; *P*-value = .023) were identified as independent risk factors (Table 5).

**Table 5.** Comparison of Demographics, Comorbidities, Hematological Conditions and Other Predisposing Factors Between NTM Disease Cases and Matched Controls

Variable	Cases (*n* = 23)	Controls (*n* = 46)	Unadjusted OR (95% CI)	*P* Value^a^	Adjusted OR (95% CI)	*P* Value^a^
Age at diagnosis, years [mean ± SD]	48.4 ± 17.6	50.0 ± 17.5	0.99 (0.97–1.02)^b^	.789	…	…
Male gender [*n* (%)]	14 (60.9)	27 (58.7)	1.06 (0.46–2.45)	.887	…	…
Chronic lung condition [*n* (%)]	7 (30.4)	3 (6.5)	2.58 (1.06–6.27)	.036	1.59 (0.63–4.06)	.325
Diabetes mellitus [*n* (%)]	5 (21.7)	4 (8.7)	1.85 (0.69–4.99)	.223	…	…
Obesity [*n* (%)]	3 (13.0)	7 (15.2)	0.89 (0.26–2.98)	.844	…	…
CMV disease [*n* (%)]^c^	3 (13.0)	2 (4.3)	1.92 (0.57–6.46)	.292	…	…
Infections other than NTM disease [*n* (%)]^d^	16 (69.6)	11 (23.9)	3.56 (1.46–8.64)	.005	3.11 (1.25–7.78)	.015
Active chemotherapy [*n* (%)]^d^	4 (17.4)	5 (10.9)	1.40 (0.48–4.13)	.538	…	…
Parenteral nutrition [*n* (%)]^d^	3 (13.0)	2 (4.3)	1.92 (0.57–6.46)	.292	…	…
Previous ICU admission [*n* (%)]^d^	5 (21.7)	0 (0.0)	3.57 (1.33–9.60)	.012	NA^f^	NA^f^
Unrelated allogeneic HSCT [*n* (%)]^e^	17 (73.9)	19 (41.3)	2.59 (1.02–6.59)	.044	1.83 (0.70–4.76)	.215
Myeloablative conditioning regimen [*n* (%)]	17 (73.9)	42 (91.3)	0.48 (0.19–1.22)	.122	…	…
Underlying hematological condition [*n* (%)]	…	…	…	…	…	…
AML	6 (26.1)	18 (39.1)	0.66 (0.26–1.68)	.385	…	…
MDS	6 (26.1)	3 (6.5)	2.35 (0.93–5.97)	.072	…	…
B-cell leukemia or lymphoma	4 (17.4)	10 (21.7)	0.83 (0.28–2.43)	.730	…	…
MM	2 (8.7)	5 (10.9)	0.84 (0.19–3.59)	.818	…	…
Non-malignant condition	3 (13.0)	4 (8.7)	1.33 (0.39–4.47)	.646	…	…
Relapsed disease after HSCT [*n* (%)]	3 (13.0)	10 (21.7)	0.65 (0.19–2.17)	.481	…	…
GVHD [*n* (%)]	20 (87.0)	30 (65.2)	2.53 (0.75–8.53)	.133	…	…
Immunosuppressive therapy [*n* (%)]^d^	20 (87.0)	28 (60.9)	2.92 (0.87–9.82)	.084	…	…
Calcineurin inhibitor [*n* (%)]^d^	13 (56.5)	25 (54.3)	1.06 (0.47–2.42)	.889	…	…
Ruxolitinib [*n* (%)]^d^	6 (26.1)	1 (2.2)	3.13 (1.23–7.93)	.016	NA^f^	NA^f^
Systemic corticosteroids [*n* (%)]^d^	16 (69.6)	12 (26.1)	3.35 (1.38–8.14)	.008	2.88 (1.16–7.17)	.023

AML, acute myelogenous leukemia; BMI, body mass index; CI, confidence interval; CMV, cytomegalovirus; GVHD, graft-versus-host disease; HSCT, hematopoietic stem-cell transplantation; ICU, intensive care unit; MDS, myelodysplastic syndrome; MM, multiple myeloma; NA, not applicable; NTM, non-tuberculous mycobacteria; OR, odds ratio; SD, standard deviation.

^a^Bold characters denote *P*-values <.005.

^b^OR per 1-year increment.

^c^Within 180 days prior to diagnosis (or the corresponding “pseudodate” of diagnosis for controls).

^d^Within 90 days prior to diagnosis.

^e^With related allogeneic or autologous GVHD as the reference category.

^f^The variables “previous ICU admission” and “ruxolitib treatment” were excluded from the multivariable model due to collinearity.

## DISCUSSION

In the present study we found that NTM disease typically occurs late after HSCT in patients with AML as the underlying condition, GVHD and ongoing immunosuppression with ruxolitinib and corticosteroids. Taken together, these findings suggest that this complication mainly affects to heavily immunocompromised patients. Although attributable death occurred in <5% of cases, the notable all-cause mortality rate at year one and the independent associations found with previous non-NTM infection and corticosteroid therapy suggest that NTM disease would act as a marker of the net state of immunosuppression.

Two-thirds of the cases were due to MAC and involved the respiratory tract in the form of nodules and interstitial infiltrates. A recent meta-analysis pooled data from 56 case reports and cohort studies published between 1983 and 2020. In line with our results, the majority of adult patients had non-disseminated infection with pulmonary involvement and MAC as the most common species, although *M. haemophilum* was predominant in cutaneous forms [18]. Of note, NTM-attributable mortality rate reported in this systematic review (33%) was notably higher than that observed in our experience. In a single-center retrospective study performed between 1995 and 2002 in China, one-fifth of patients died with persistently positive acid-fast bacilli (AFB) staining and cultures despite receiving antimycobacterial treatment [23]. Another study reported a NTM-attributable mortality between 2003 and 2014 of 11.5% [8]. Beyond the inherent difficulties in the attribution of the cause of death, these findings would point to improved outcomes over time, even though there have been few changes in guideline-based treatment recommendations.

The most common antimycobacterial regimen used in our study included macrolides, rifamycins and ethambutol, whereas the median duration of therapy was similar to that reported in the literature [18]. It may be hypothesized that the reduction or withdrawal of concurrent immunosuppression, which was performed in one quarter of the cases, may have contributed to the relatively better outcome in comparison to older series [8]. The predominance in our experience of cases of fibronodular pulmonary MAC infection —with <10% of patients with disseminated disease—also confers a more favorable outcome as compared to previous reports with higher rates of disseminated forms (20.0% in a pediatric cohort [20], 25.0% in a series of catheter-related BSI [15] or 21.7% in the systematic review by Cinicola et al [18]). Nevertheless, the proportion of patients that required discontinuation of therapy due to treatment-related AEs was higher than in the SOT recipients included in the EMOTE study [25].

The predominance of pulmonary disease has been also observed in previous series of adult HSCT recipients [16, 21, 23] and aligns with the overall non-transplant population. Interestingly, the presence of cavitation or multifocal bronchiectasis in thoracic CT scans was rare, in contrast with the Lady Windermere syndrome described in middle-aged women with MAC infection and middle lobe and lingular bronchiectasis [2–5]. We identified three cases of catheter-related BSI, all of them due to RGM (*M. mucogenicum* in two patients and *M. fortuitum* in the remaining one). In the literature review by Nagata et al, where RGM accounted for almost 90% of cases of NTM BSI, there was an overall favorable outcome provided that a combination of at least two active agents was administered for a minimum of 4 weeks in addition to catheter removal [15]. Accordingly, NTM BSI cases in our cohort were all due to RGM and treated for a median of 29 days only.

Some authors have previously investigated the risk factors for NTM disease after HSCT. Beswick et al. [21] identified the severity of chronic GVHD and CMV DNAemia, and proposed a risk score based on both variables that allowed to identify allo-HSCT recipients at intermediate and high risk for this complication [21]. The results of our study point in a similar direction, since it is plausible that the development of other non-NTM infections—specifically CMV DNAemia—may act as a marker of delayed immune reconstitution. Antimicrobial-induced dysbiosis or severe infections may lead to a transient state of immune exhaustion and impaired antimycobacterial immunity [27, 28]. Finally, it cannot be completely ruled out that previous non-NTM infection might have actually represented a missed diagnosis of NTM disease. These infections can be difficult to diagnose due to the non-specific clinical presentation and the lack of mycobacterial culture of clinical samples. A retrospective study carried out in Japan revealed that one-third of cases remained undiagnosed for 1 year from the first visit [29]. Misdiagnosis with tuberculosis is not uncommon in high-burden countries [30]. In addition, it has been reported that some NTM species may be misdiagnosed as *M. tuberculosis* by the GeneXpert MTB/RIF assay in samples with high bacterial load [31, 32]. Unfortunately, preceding diagnoses of non-NTM infection could not be reviewed due to the retrospective design of our research.

Au et al. found that HLA mismatched or unrelated donor allo-HSCT—which closely correlates with the occurrence of GVHD [33]— acted as the only independent predictor of NTM infection [23]. The effect of GVHD in that study may be attributable to the concomitant use of systemic corticosteroids. Although the association was no longer significant after multivariable adjustment, the identification of chronic pulmonary condition as a risk factor in the present experience is consistent with previous studies performed in the HSCT setting [23] and the general population [34].

Our study has several limitations, mainly derived from its retrospective nature and the low number of cases of NTM included despite the participation of 23 centers, which exemplifies the challenges to investigate this uncommon infection. The low sample size may have affected the stability of the multivariable model, with the associated risk of overfitting. We lacked details on the type and severity of GVHD. Although standardized definitions and consensus criteria were applied, we cannot rule out that inaccurate or missing data and heterogeneous diagnostic practices across participating centers may have resulted in information bias. Also inherent to the retrospective design, the methodology for NTM species identification (e.g., biochemical assays, molecular methods or MALDI-TOF) was not standardized across centers. Finally, the case-control design did not allow for any estimation regarding incidence or prevalence of NTM disease in the HSCT population. On the other hand, the external validity of our research is strengthened by the inclusion of a control group, the multinational collaboration and the recent recruitment period. It should be noted, however, that the matching was performed on general, non-specific factors (center and post-transplant survival) due to the inherent heterogeneity of sporadic cases of NTM disease.

In conclusion, NTM are potentially severe opportunistic pathogens in HSCT recipients, mainly in the form of pulmonary disease due to MAC. Although attributable mortality seems to be lower than previously reported, the occurrence of toxicity related to antimycobacterial therapy is significant in this population. Patients with impaired immunity, as reflected by previous non-NTM infection and recent or ongoing corticosteroid therapy, appear to be at increased risk and would benefit the most from maintaining a high index of suspicion and low threshold for diagnostic testing based on proper methods (e.g., AFB staining, mycobacterial culture and molecular identification).

## Supplementary Material

ofag082_Supplementary_Data

## Supplementary Data

Supplementary materials are available at *Open Forum Infectious Diseases* online. Consisting of data provided by the authors to benefit the reader, the posted materials are not copyedited and are the sole responsibility of the authors, so questions or comments should be addressed to the corresponding author.
