# Supplementary material for: Clinical Presentation, Risk Factors and Outcome of Non-Tuberculous Mycobacteria Infection in Hematopoietic Stem-Cell Transplantation: A Multinational Case-Control Study
Source: Open Forum Infect Dis. 2026 Feb 19;13(2):ofag082. doi: 10.1093/ofid/ofag082 (PMC12934346; doi:10.1093/ofid/ofag082)
Supplement: ofag082_Supplementary_Data [file ofag082_supplementary_data.docx]

***Supporting Material***

**Supplementary Results**

**Table S1.** Individual details on clinical presentation, radiological features and outcomes in cases of NTM disease.

| **Case** | **Age, gender (years)** | **Underlying disease** | **Type of HSCT** | **GVHD** | **NTM species** | **Site of infection** | **Pulmonary involvement in CT scan at diagnosis** | **Outcome** |
| --- | --- | --- | --- | --- | --- | --- | --- | --- |
| 1 | Female, 60.2 | MDS | Allo, unrelated | Yes | MAC | Pulmonary | Alveolar infiltrate | Alive, no infection relapse |
| 2 | Female, 58.2 | Aplastic anemia | Allo, unrelated | Yes | MAC | Cutaneous | - | Alive, no infection relapse |
| 3 | Male, 65.3 | Myeloma | Auto | No | MAC | Disseminated | - | Relapse |
| 4 | Male, 51.2 | CML | Allo, related | Yes | MAC | Cutaneous | - | Alive, no infection relapse |
| 5 | Female, 52.9 | MDS | Allo, unrelated | Yes | MAC | Pulmonary | Nodules, bronchiectasis | Alive, no infection relapse |
| 6 | Female, 52.1 | AML | Allo, unrelated | Yes | MAC | Lymphadenitis | - | Alive, no infection relapse |
| 7 | Male, 62.1 | AML | Allo, unrelated | Yes | MAC | Pulmonary | Nodules, pleural effusion | Attributable death |
| 8 | Male, 57.6 | NHL | Allo, related | Yes | MAC | Pulmonary | Nodules, interstitial infiltrate | Alive, no infection relapse |
| 9 | Female, 26.0 | Blastic dendritic cell neoplasm | Allo, unrelated | Yes | MAC | Pulmonary | Nodules, pleural effusion | Alive, no infection relapse |
| 10 | Female, 22.9 | AML | Allo, unrelated | Yes | MAC | Pulmonary | Alveolar infiltrate | Alive, no infection relapse |
| 11 | Male, 44.5 | CVID | Allo, unrelated | No | MAC | Pulmonary | Nodules | Alive, no infection relapse |
| 12 | Male, 59.5 | MDS | Allo, unrelated | Yes | MAC | Pulmonary | Nodules | Attributable death |
| 13 | Male,61.8 | MDS | Allo, unrelated | Yes | MAC | Pulmonary | Interstitial infiltrate | Attributable death |
| 14 | Male, 16.3 | AML | Allo, related | Yes | MAC | Pulmonary | Alveolar infiltrate | Attributable death |
| 15 | Female, 13.5 | CTLA-4 deficiency | Allo, unrelated | Yes | MAC | Pulmonary | Interstitial and alveolar infiltrates, cavitation | Alive, no infection relapse |
| 16 | Female, 60.7 | Myeloma | Auto | No | MAC | Pulmonary | Nodules | Alive, no infection relapse |
| 17 | Male, 54.7 | AML | Allo, unrelated | Yes | *M. chelonae-abscessus* | Pulmonary | Nodules, interstitial infiltrate | Attributable death |
| 18 | Male, 28.4 | AML | Allo, related | Yes | *M. chelonae-abscessus* | Pulmonary | Interstitial infiltrate | Attributable death |
| 19 | Male, 68.1 | CLL | Allo, unrelated | Yes | *M. chelonae-abscessus* | Pulmonary | Nodules | Alive, no infection relapse |
| 20 | Male, 30.3 | MDS | Allo, unrelated | Yes | *M. fortuitum* | BSI | - | Alive, no infection relapse |
| 21 | Female, 56.5 | AML | Allo, unrelated | Yes | *M. mucogenicum* | BSI | - | Alive, no infection relapse |
| 22 | Female, 65.9 | MDS | Allo, unrelated | Yes | *M. mucogenicum* | BSI | - | Alive, no infection relapse |
| 23 | Male, 66.0 | NHL | Allo, unrelated | Yes | *M. kansasii* | Pulmonary | Interstitial and alveolar infiltrates | Attributable death |
| 24 | Male, 31.2 | ALL | Allo, unrelated | Yes | *M. kansasii* | Disseminated | Interstitial and alveolar infiltrates | Attributable death |
| 25 | Male, 68.0 | NHL | Allo, related | No | *M. lentiflavum* | Pulmonary | Interstitial infiltrate | Alive, no infection relapse |
| Allo: allogeneic; ALL: acute lymphoblastic leukaemia; AML: acute myelogenous leukemia; BSI: bloodstream infection; CML: chronic myelogenous leukemia; CT: computed tomography; CVID: common variable immunodeficiency; GVHD: graft-versus-host disease; HSCT: hematopoietic stem-cell transplantation; MAC: *Mycobacterium avium* complex; MDS: myelodysplastic syndrome; NHL: non-Hodgkin lymphoma; NTM: non-tuberculous mycobacteria. | | | | | | | | |

**Table S2.** *In vitro* antimicrobial susceptibly testing according to the CLSI methodology.

| **NTM species** | **Antimycobacterial agent (no. of tested isolates)** | **CLSI category** | | |
| --- | --- | --- | --- | --- |
|  |  | Sensitive | Intermediate | Resistant |
| MAC | Amikacin (n = 7) | 0 / 7 | 2 / 7 | 5 / 7 |
|  | Clarithromycin (n = 12) | 12 / 12 | 0 / 12 | 0 / 12 |
|  | Ethambutol (n = 9) | 2 / 9 | 1 / 9 | 6 / 9 |
|  | Linezolid (n = 7) | 1 / 7 | 2 / 7 | 4 / 7 |
|  | Moxifloxacin (n = 4) | 1 / 4 | 1 / 4 | 2 / 4 |
|  | Rifampicin (n = 8) | 0 / 8 | 1 / 8 | 7 / 8 |
|  | Rifabutin (n = 6) | 3 / 6 | 1 / 6 | 2 / 6 |
| RGM | Amikacin (n = 6) | 6 / 6 | 0 / 6 | 0 / 6 |
|  | Cefoxitin (n = 6) | 3 / 6 | 3 / 6 | 0 / 6 |
|  | Ciprofloxacin (n = 6) | 3 / 6 | 3 / 6 | 0 / 6 |
|  | Clarithromycin (n = 6) | 4 / 6 | 0 / 6 | 2 / 6 |
|  | Doxycicline (n = 6) | 2 / 6 | 0 / 6 | 4 / 6 |
|  | Linezolid (n = 6) | 4 / 6 | 1 / 6 | 1 / 6 |
| CLSI: Clinical and Laboratory Standards Institute; MAC: *Mycobacterium avium* complex RGM: rapidly growing mycobacteria. | | | | |
